# A direct measurement method of quantum relaxation time

**DOI:** 10.1093/nsr/nwaa242

**Published:** 2020-09-18

**Authors:** Peng Zhang, Haoqi Tang, Chuanchuan Gu, Hong Wang, Guangfu Luo, Yalin Lu, X-D Xiang

**Affiliations:** Department of Materials Science and Engineering & Department of Physics, Southern University of Science and Technology, Shenzhen 518055, China; Department of Materials Science and Engineering & Department of Physics, Southern University of Science and Technology, Shenzhen 518055, China; Department of Materials Science and Engineering & Department of Physics, Southern University of Science and Technology, Shenzhen 518055, China; Synergetic Innovation Center of Quantum Information and Quantum Physics & Hefei National Laboratory for Physical Sciences at Microscale, University of Science and Technology of China, Hefei 230026, China; Materials Genome Initiative Center and School of Materials Science and Engineering, Shanghai Jiao Tong University, Shanghai 200240, China; State Key Laboratory of Green Building Materials, China Building Materials Academy, Beijing 100024, China; Department of Materials Science and Engineering & Department of Physics, Southern University of Science and Technology, Shenzhen 518055, China; Guangdong Provincial Key Laboratory of Computational Science and Material Design, Southern University of Science and Technology, Shenzhen 518055, China; Synergetic Innovation Center of Quantum Information and Quantum Physics & Hefei National Laboratory for Physical Sciences at Microscale, University of Science and Technology of China, Hefei 230026, China; Department of Materials Science and Engineering & Department of Physics, Southern University of Science and Technology, Shenzhen 518055, China

**Keywords:** quantum relaxation time, plasma resonance, dielectric loss function

## Abstract

The quantum relaxation time of electrons in condensed matters is an important physical property, but its direct measurement has been elusive for a century. Here, we report a breakthrough that allows direct determination of quantum relaxation time at zero and non-zero frequencies using optical measurement. Through dielectric loss function, we connect bound electron effects to the physical parameters of plasma resonance and find an extra term of quantum relaxation time from inelastic scattering between bound electrons and conduction electrons at non-zero frequencies. We demonstrate here that the frequency-dependent inelastic polarization effect of bound electrons is the dominant contribution to quantum relaxation time of conduction electrons at optical frequencies, and the elastic polarization effect of bound electrons also dramatically changes the plasma resonance frequency through effective screening to charge carriers.

## Teaser

Taking new mechanism of conducting electrons-bound electrons into account, quantum relaxation time for the first time could be directly determined via far-field optical measurement.

## INTRODUCTION

Quantum relaxation time (*τ*) is an important physical property affecting some of the most critical electron transport parameters in advance materials, such as electrical conductivity and carrier mobility in metals and semiconductors, the pseudo-gap and critical temperature of superconductors [1], and the propagation distance of an electron carrying encoded information in quantum computation materials and devices [2,3]. It also relates to the weak localization effect of topological materials [4,5] and the coupling of multi-degrees of freedom in strongly correlated systems [6]. In optical-driven electronic devices [7–9], *τ* under an electromagnetic (EM) field is a critical factor in determining the information exchange between electrons and photons.

Measurement of *τ* has never been a straightforward task. Conventionally, *τ* can only be determined indirectly under a static field by the equation: *τ* = μm^*^/*e*. While the carrier mobility (*μ*) is identified by joint measurements of DC conductivity and Hall effect using electrical-contact methods, the effective mass (m^*^) is obtained by magnetic oscillation experiments under an ultra-high magnetic field and ultra-low temperature. A method for direct measurement of quantum relaxation time is a constant pursuit for physicists, especially at non-zero frequencies.

In 1900, Drude proposed a theory describing the interaction between photons and conduction electrons [10], which paved the way towards an optical solution for quantum relaxation time measurement. The Drude model predicts the resonance of conducting electrons in a material under optical radiation at plasma frequency *ω_p_.* The Drude model was later modified [11] with quantum theory by Sommerfeld and others, resulting in a universal expression of the Drude–Sommerfeld complex dielectric function for all conductive materials, that is the response of a free electron gas to an optical radiation derived from the Maxwell equation [12]:

(1) \begin{eqnarray*} {\varepsilon ^D}\! \left( {\rm{\omega }} \right) &=& \varepsilon _r^D\ \ + \ i \varepsilon _i^D = \left( {{\rm{\ }}{\varepsilon _c} - \frac{{\omega _p^2}}{{{\omega ^2} + 1/\tau _D^2}}\ } \right)\nonumber\\ &&+\, \ i\frac{{\omega _p^2}}{{\omega {\tau _D}\left( {{\omega ^2} + 1/\tau _D^2} \right)}},\end{eqnarray*}

where

(2) \begin{equation*}\omega _p^2 = \frac{{4\pi {n_e}{e^2}}}{{{m^*}}},\end{equation*}

and ${\tau _D}$ is the Drude quantum relaxation time (reciprocal of the electron scattering rate), which is considered frequency-independent and thus can be determined under DC field; ${\varepsilon _c}$ the background dielectric polarizability [13] equals unity in the original model; ${n_e}\ $the number of conduction electrons (around the Fermi surface) per unit volume; and m^*^ the effective mass of conduction electrons.

Via introduction of effective mass, the background lattice effect to the conduction electrons can be partly accounted for in Equations (1) and (2) and the plasma frequency is rewritten [14] with a redefinition of electron mass by band structure effective mass of quasi-particles. Subsequently, the single quantum relaxation time of all conduction electrons hypothesized by Drude is naturally explained by single Fermi energy as per Fermi-Dirac statistics for all conductive electrons. This has been proven valid even for a highly correlated heavy-fermion system [6].

In more recent works [15–18], a constant ${\varepsilon _c} = \ 1 + 4\pi n\alpha $ is introduced to resolve the discrepancies between the model and experimental values of plasma frequency, where $\alpha $, a constant, is the ionic polarizability coefficient, and *n* is the atomic density. As a result, the resonance is expected to occur where ${\varepsilon _r}( \omega )$ vanishes at the so-called screened plasma frequency *ω_s_* [19], instead of *ω_p_*, to account for the frequency shift from screening of conduction electrons, where

(3) \begin{equation*}\omega _s^2 = \frac{{\omega _p^2}}{{{\varepsilon _c}}}\ - \frac{1}{{\tau _D^2}}.\end{equation*}

Here, the background dielectric polarizability ${\varepsilon _c}$ for different materials is obtained by fitting the plot of $\varepsilon _r^D\ vs\ 1/{\omega ^2}$ over wide range frequencies [15–18] with the assumption that $\omega \gg 1/{\tau _D}$:

(4) \begin{equation*}\varepsilon _r^D\!\left( {\rm{\omega }} \right) \approx {\varepsilon _c} - \frac{{\omega _p^2}}{{{\omega ^2}}}.\end{equation*}

The slope and intercept of the plot correspond to $\omega _p^2$ and ${\varepsilon _c}$, respectively.

**Figure 1.**
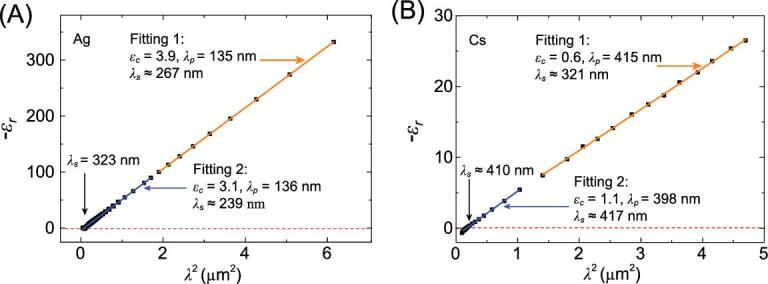
The fits of (A) Ag and (B) Cs in different ranges: the experimental data (black square) [20,21] and fitting results based on Equation (4). The fitting processes for Ag are in the range from 210 nm to 1305 nm (blue curve in (A)) and from 1350 nm to 2480 nm (orange curve in (A)); Cs are in the range from 310 nm to 1050 nm (blue curve in (B)); and from 1100 nm to 2200 nm (orange curve in (B)), respectively.

However, problems remain. Firstly, a constant value of ${\varepsilon _c}$ > 1 is not physical, as when $\omega \to \infty $, ${\varepsilon _c}$ should equal unity from both experimental and theoretical points of view. Secondly, as fitting with experimental data in different frequency ranges yields varied slopes with$\ 1/{\omega ^2}$, the resulting ${\varepsilon _c}$ cannot be uniquely determined (differing by several times). As shown in Fig. 1(A), the fits of Ag based on Equation (4) in different wavelength ranges would give varied fitting results. Moreover, the screened plasma wavelengths do not match the experimentally determined resonance frequencies. For Ag and Au, ${\lambda _s} = \frac{{2\pi c}}{{{\omega _s}}}\ $ are calculated to be 267 and 329 nm based on Equations (3) and (4) using reported data [20], which are 21–47% smaller than the observed resonance frequencies of 323 and 485 nm, respectively. Notably, deviations in fitting greatly increase in the alkali metals (Cs), as shown in Fig. 1(B).

The relaxation time ${\tau _D}$ can also be deduced by applying the same assumption to the imaginary part of Equation (1) [22,23] as

(5) \begin{equation*}\varepsilon _i^D\!\left( {\rm{\omega }} \right) \cdot \omega \approx \frac{{\omega _p^2}}{{{\omega ^2}}} \cdot \frac{1}{{{\tau _D}}}.\end{equation*}

Thèye [22] obtained the quantum relaxation time of Au films from Equation (5) using dielectric constant data over a wide frequency range, and found that $1/{\tau _D}$ is, to a certain extent, frequency-dependent. Nagel and Schnatterly [24] proposed a two-carrier model emphasizing the contribution from conduction electrons scattering and disordering in grain boundaries. However, the reciprocal relaxation time from the disorder effect increases only slightly (< 10%) with frequency for annealed samples, far from being sufficient to account for the experimentally observed 5-fold increase in $1/{\tau _D}$ at plasma resonance [17,18,20,21,25].

Thus, as shown in the above examples, there exists a large deficiency for the Drude–Sommerfeld model to explain the experimental data. The dielectric function is a consequence of the primary effect from interaction between the EM field and free electrons correctly described by the Drude model, characterized by the bare plasma frequency *ω_p_* and the frequency-independent quantum relaxation time ${\tau _D}$; it is also a consequence of the primary effect from interaction between the EM field and bound electrons, resulting from excitations or transitions from the valence band to the conduction band. We believe that a secondary effect, which results from interaction between conducting electron oscillation and bound electron oscillation, accounts for the large deficiency between the description of the Drude model and optical data.

**Figure 2.**
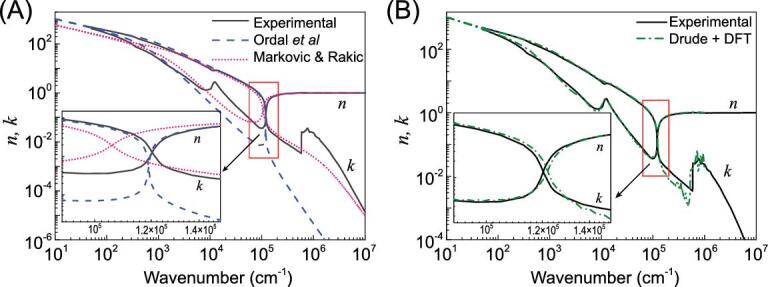
(A) Comparison of experimental and calculated values of *n*(*ω*), *k*(*ω*) for aluminum: experimental data [26] (black solid line), Drude results based on Equation (1) by Ordal *et al.* [27] (blue dashed line) and Drude model modified with a square-frequency dependence of reciprocal relaxation time by Markovic and Rakic [26] (pink dotted line). (B) Experimental data [26] (black solid line) and Drude model (parameters in Table 1) combined with DFT calculations (green dash-dotted line).

Therefore, the bound electron effect must be included in the model analysis. With this in mind, the total complex dielectric function should be written as [12,19]:

(6) \begin{equation*}\varepsilon\! \left( {\rm{\omega }} \right) = {\varepsilon ^D}\! \left( \omega \right) + {\varepsilon ^B}\!\left( \omega \right),\end{equation*}

where ${\varepsilon ^B}\! ( {\rm{\omega }} ) = \varepsilon _r^B\! ( {\rm{\omega }} ) + i\varepsilon _i^B\!( {\rm{\omega }} )$ describes the elastic and inelastic deformation of the bound electron polarization effect and can be calculated according to Fermi's golden rule through density functional theory. The Lorentz simple harmonic oscillator model was used to approximate ${\varepsilon ^B}( \omega )$ [12,19], but success was limited. In another attempt, Markovic and Rakic [26] proposed inclusion of a frequency-dependent `electron re-radiation' effect in the Drude–Sommerfeld model, related to response to the EM wave from both conduction and bound electrons, and causing a change of phase speed of EM radiation. By replacing $1/{{\rm{\tau }}_{\rm{D}}}$ with $1/{\rm{\tau }}( {\rm{\omega }} ){\rm{\ }} = {\rm{\ }}1/{{\rm{\tau }}_{\rm{D}}} + {\rm{b}}{\omega ^2}$, the complex refractive index [*n*(*ω*) and *k*(*ω*)] of Al is fitted as shown in Fig. 2(A). Not only does the fitted plasma frequency (∼94 nm) deviate from the experimental data (83 nm) more than that obtained from the simple Drude–Sommerfeld model [27], but also the reciprocal relaxation time (1590 cm^−1^) at the plasma resonance frequency is three times the DC value of ∼550 cm^−1^ (from DC resistivity [28] and ${\omega _p}$ by $\frac{1}{{{\tau _D}}} = \frac{{\omega _p^2}}{{4\pi }}\ {\rho _{dc}}$), dramatically deviating from the experimental results.

It comes to our realization that ${\varepsilon ^B}( \omega )$ is a very complex and sample-dependent term, which requires a more complex function of superposition of multiple harmonic oscillators. In Fig. 2(B), we calculate the *n*(*ω*) and *k*(*ω*) of Al based on Equation (6) with ${\varepsilon ^B}( \omega )$ obtained by density functional theory (DFT) [29], which takes into account all possible band transitions and appropriate quantum statistics. Compared with the data in Fig. 2(A), a significant improvement is achieved with a more suitable bound electron term on the dielectric function. This proves that for the model to match with the optical data, a complex form must be used to describe the bound electron effect with sufficient detail.

In this work, we propose a new measuring method accounting for contributions of both conduction (Drude term) and bound electrons to determine frequency-dependent quantum relaxation times. We subsequently analyze the complex bound electron effects with experimental data through multi-parameter fitting of dielectric loss function. The results clearly prove that bound electrons play a dominant role in quantum relaxation at optical frequencies.

## RESULTS

To understand the impact of the bound electron term ${\varepsilon ^B}( \omega )$ on the damping effect to conduction electrons at plasma resonance, we hereby propose adopting an approach used for electron scattering loss analysis [12]. First, we choose to utilize the dielectric loss function (DLF, defined as the inverse of the dielectric function)

(7) \begin{equation*}\frac{1}{{\varepsilon\! \left( {\rm{\omega }} \right)}} = \frac{{{\varepsilon _r}\!\left( \omega \right) - i{\varepsilon _i}\!\left( \omega \right)}}{{\varepsilon _r^2\!\left( \omega \right) + \varepsilon _i^2\!\left( \omega \right)}}.\end{equation*}

If only considering the interaction with free electrons ${\varepsilon ^D}( \omega )$, the real and imaginary parts of DLF $\frac{1}{{\varepsilon ( {\rm{\omega }} )}}$ are given by Dressel and Gruner [12] as

$$\begin{equation*}
{\rm{Re}}{\left\{ {\frac{1}{{\varepsilon\! \left( \omega \right)}}} \right\}_{\rm{D}}} = {\rm{\ }}1 + \frac{{\left( {{\omega ^2} - \omega _p^2} \right)\omega _p^2}}{{{{\left( {{\omega ^2} - \omega _p^2} \right)}^2} + {\omega ^2}\tau _D^{ - 2}}},\end{equation*}$$

and

**Figure 3.**
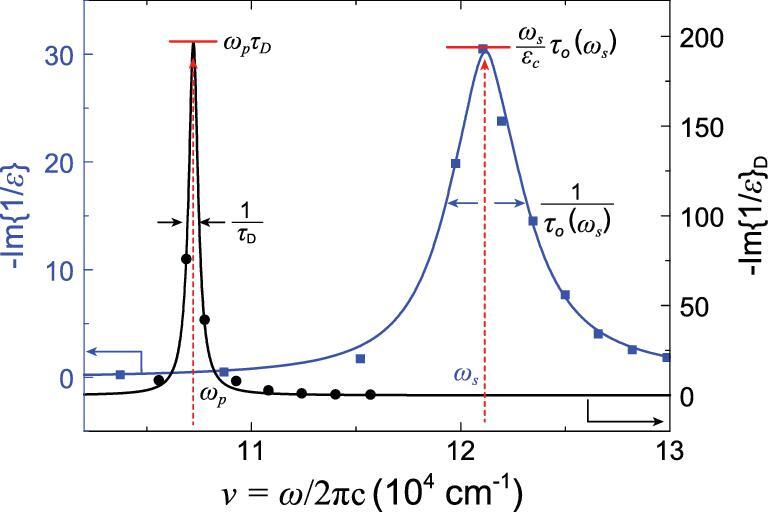
The imaginary part of Drude (black line) and complete (blue line) dielectric loss function of Al [25] with parameters of ${\varepsilon _c} = \ 0.78$, ${\omega _p}/( {2\pi c} )\ = \ 106\;873\ {\rm{c}}{{\rm{m}}^{ - 1}}$, ${\varepsilon ^B}\ ( {{\omega _s}} ) = \ - 0.22 + i\ 0.028$. The blue squares are experimental values, while the black dots are the corresponding Drude-only results based on the analysis of ${\varepsilon ^D}\! ( \omega ) = \ \varepsilon ( {\rm{\omega }} ) - {\varepsilon ^B}( \omega )$.

(8) \begin{equation*} -\! {\rm{Im}}{\left\{ {\frac{1}{{\varepsilon \left( \omega \right)}}} \right\}_{\rm{D}}} = \frac{{\omega _p^2\omega /{\tau _D}}}{{{{\left( {{\omega ^2} - \omega _p^2} \right)}^2} + {\omega ^2}\tau _D^{ - 2}}},\end{equation*}

respectively. As shown in Fig. 3, the Drude term $ - {\rm{Im}}{\{ {\frac{1}{{\varepsilon ( \omega )}}} \}_{\rm{D}}}$ has a very sharp symmetric plasma resonance peak at ${\omega _p}$, with a maxima of ${\omega _p}{\tau _D}$, and full width at half maximum (FWHM) of $1/{\tau _D}$. Considering the secondary scattering effect between conducting and bound electrons, we include the bound electron effect described by ${\varepsilon ^B}$ in the DLF. The resonance frequency shifts from ${\omega _p}$ to the screened plasma frequency ${\omega _s}$, given by

(9) \begin{equation*}\omega _s^2 = \frac{{\omega _p^2}}{{{\varepsilon _c}\!\left( {{\omega _s}} \right)}}\ - \frac{1}{{\tau _D^2}},\end{equation*}

(10) \begin{equation*}{\varepsilon _c}\! \left( {{\omega _s}} \right) = \ 1 + \varepsilon _r^B\! \left( {{\omega _s}} \right).\end{equation*}

Here ${\varepsilon _c}( {{\omega _s}} )$ is not an arbitrary number, but a measurable and calculable physical quantity that approaches 1 as $\omega \to \infty $. As ${\omega _s}$ depends on carrier density ${n_e}$, in principle it can be controlled to be any frequency, especially through impurity or optical doping levels in semiconducting materials. Therefore, Equation (10) is valid for any frequency.

Taking ${\varepsilon _r}\! ( \omega ) = \varepsilon _r^D\! ( \omega ) + \varepsilon _r^B( {\rm{\omega }} )\ {\rm{and}} {\varepsilon _i}\! ( \omega ) = \varepsilon _i^D\! ( \omega ) + \varepsilon _i^B( {\rm{\omega }} )$ into Equation (7) yields

$$\begin{equation*}{\rm{Re }}\left( {\frac{1}{{\varepsilon\ \left( \omega \right)}}} \right) = \frac{{1 - \frac{{\omega _p^2}}{{{\omega ^2} + \tau _D^{ - 2}}} + \varepsilon _r^B\!\left( {\rm{\omega }} \right)}}{{{{\left( {1 - \frac{{\omega _p^2}}{{{\omega ^2} + \tau _D^{ - 2}}} + \varepsilon _r^B\!\left( {\rm{\omega }} \right)} \right)}^2} + {{\left( {\frac{{\omega _p^2}}{{\omega {\tau _D}\left( {{\omega ^2} + \tau _D^{ - 2}} \right)}} + \varepsilon _i^B\!\left( {\rm{\omega }} \right)} \right)}^2}}},\end{equation*}$$

and

(11) \begin{equation*} {\rm{Im }}\left( {\frac{1}{{\varepsilon \left( \omega \right)}}} \right) = \frac{{\frac{{\omega _p^2}}{{\omega {\tau _D}\left( {{\omega ^2} + \tau _D^{ - 2}} \right)}} + \varepsilon _i^B\!\left( {\rm{\omega }} \right)}}{{{{\left( {1 - \frac{{\omega _p^2}}{{{\omega ^2} + \tau _D^{ - 2}}} + \varepsilon _r^B\!\left( {\rm{\omega }} \right)} \right)}^2} + {{\left( {\frac{{\omega _p^2}}{{\omega {\tau _D}\left( {{\omega ^2} + \tau _D^{ - 2}} \right)}} + \varepsilon _i^B\!\left( {\rm{\omega }} \right)} \right)}^2}}}. \end{equation*}

Using ${\varepsilon _r}\! ( {{\omega _s}} ) = \ 0$ in Equation (11), we deduce the peak value of the dielectric loss spectrum at ${\omega _s}$:

(12) \begin{equation*}-\! {\rm{Im }}\left\{ {\frac{1}{{\varepsilon \left( {{\omega _s}} \right)}}} \right\} = \frac{1}{{{\varepsilon _i}\left( {{\omega _s}} \right)}}\ = \frac{{\frac{{{\omega _s}}}{{{\varepsilon _c}\left( {{\omega _s}} \right)}}}}{{\frac{1}{{{\tau _D}}} + \frac{{\varepsilon _i^B\left( {{\omega _s}} \right){\omega _s}}}{{{\varepsilon _c}\left( {{\omega _s}} \right)}}}}.\end{equation*}

Letting Equation (12) be $\frac{{{\omega _s}}}{{{\varepsilon _c}( {{\omega _s}} )}}{\tau _o}( {{\omega _s}} )$, we obtain an equivalent optical quantum relaxation time ${\tau _o}( \omega )$ and the corresponding FWHM of this new resonance to be

(13) \begin{equation*}\frac{1}{{{\tau _o}\!\left( {{\omega _s}} \right)}} = \frac{1}{{{\tau _D}}} + \frac{1}{{{\tau _{AC}}\!\left( {{\omega _s}} \right)}},\end{equation*}

where the term $1/{\tau _{AC}}( {{\omega _s}} )\ = \varepsilon _i^B( {{\omega _s}} ){\omega _s}/{\varepsilon _c}\! ( {{\omega _s}} )$ turns the sharp symmetric resonance peak into a broadened asymmetric resonance peak as a result of inelastic scattering of conduction electrons by bound electrons, as shown in Fig. 3. Hence, measurement of the FWHM of the dielectric loss peak could provide a direct means to identify the quantum relaxation time at a given non-zero frequency (i.e. the plasma frequency).

**Figure 4.**
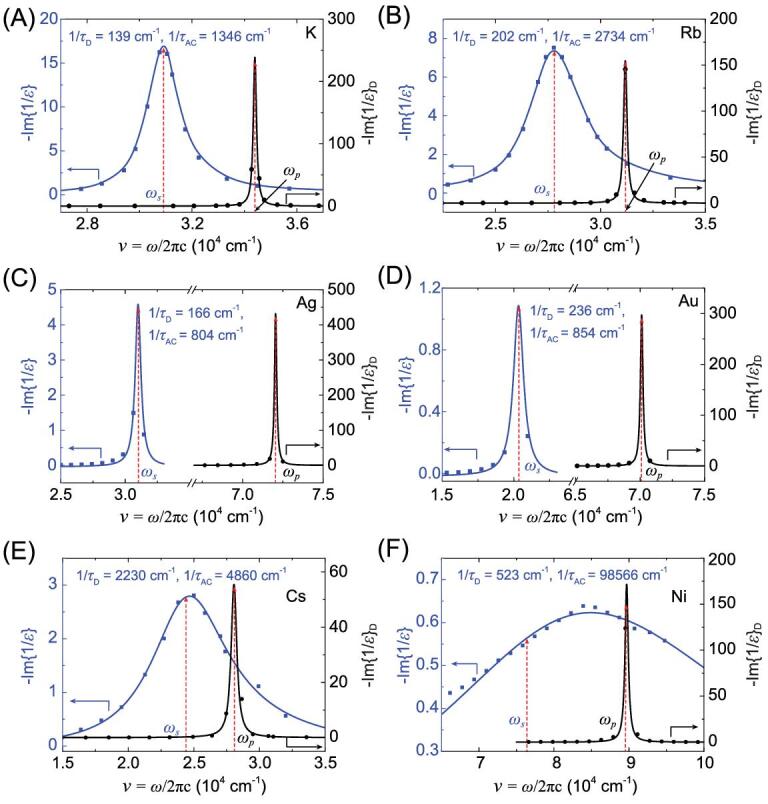
Fittings of the imaginary part of dielectric loss function based on the optical data of (A) potassium [17], (B) rubidium [18], (C) silver [20], (D) gold [20], (E) cesium [21] and (F) nickel [30]. The corresponding fitted parameters are shown in Table 1.

For a real material, we can first determine ${\omega _s}$, ${\varepsilon _c}( {{\omega _s}} )$, and ${\tau _o}( {{\omega _s}} )$ with the measured peak position, peak value and FWHM of plasma resonance, and then $\varepsilon _i^B( \omega )$ and ${\tau _D}$ by fitting the asymmetric function of $ - {\rm{Im}}\{ {\frac{1}{{\varepsilon ( \omega )}}} \}$ with optical data, as well as ${\omega _p}$ based on Equation (9) (for details of the data processing procedure, see Supplementary data S1). To manifest the Drude term, we make an axis transformation to eliminate the contribution of bound electrons (black dot and line in Figs 3 and 4), including two parts: the first is the screening of conduction carrier density, resulting in the change in plasma frequency; the second is the asymmetric broadening of the plasma resonance peak.

It is important to note that the quantum relaxation time obtained by our method is the only direct measurement to our knowledge. It is also important to note that, in addition to phonon-electron, impurity-electron and electron-electron scattering, we find, for the first time, an additional scattering mechanism for the quantum relaxation time of conduction electrons in solids at non-zero frequencies.

**Figure 5.**
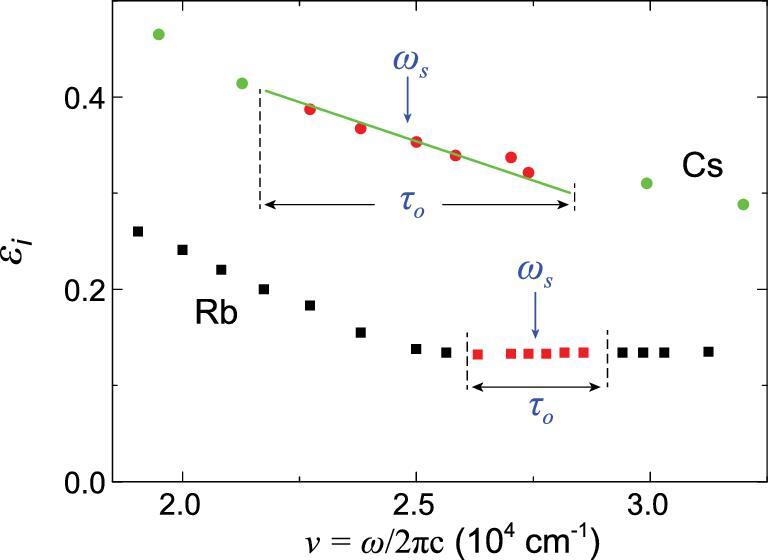
The imaginary parts of dielectric function of Rb [18] and Cs [21].

DLF analysis with bond electron contributions (DLF-BE) was performed on metals K, Rb, Ag, Au, Cs and Ni [17,18,20,21,30] and the imaginary parts $ - {\rm{Im}}\{ {\frac{1}{{\varepsilon ( \omega )}}} \}$ and $ - {\rm{Im}}{\{ {\frac{1}{{\varepsilon ( \omega )}}} \}_{\rm{D}}}$ are plotted in Fig. 4(A–F). It is noted that the ${\omega _s}$ of all six metals is red-shifted relative to ${\omega _p}$, opposite to the case of Al (Fig. 3) in which the shift is toward blue. This is attributed to the fact that the real part of dielectric function for bound electrons $\varepsilon _r^B( {{\omega _s}} )$ of Al is negative, whereas those for the other seven metals are positive (see Supplementary data S2). Within the narrow vicinity of plasma resonance, $\varepsilon _i^B( \omega )$ can be approximated as either a constant or a simple function, such as a linear function, depending on the characteristics of the measured data. For example, as shown in Fig. 5, values of $\varepsilon _i^B( \omega )$ of Rb hardly change within the plasma resonance region, and, therefore, we can assume a constant value for the fitting purpose. The $\varepsilon _i^{B^\prime}$ of Al, K, Ag, Au are also treated as constant around ${\omega _s}$. In contrast, the data for Cs [21] follow a straight line, and, thus, we fit the data with a linear function $\varepsilon _i^B\ ( \omega ) = {\rm{\ }}a + b\omega $ to yield *a* = 0.56, *b* = $ - $1.28 $ \times $ 10^−5^ cm, and the values of $\varepsilon _i^B\ ( {{\omega _s}} ) = \ 0.25$ and $1/( {2{\rm{\pi c}}{\tau _D}} ){\rm{\ }} = \ 2230 \pm 100 {\,\,\rm{c}}{{\rm{m}}^{ - 1}}$. If a constant $\varepsilon _i^B( \omega )$ is used instead of a linear function for Cs, the fitting error of $1/( {2{\rm{\pi c}}{\tau _D}} )$ would increase significantly from <10% to 50%. For Ni, a linear and parabolic function $\varepsilon _i^B(\omega ) = a + b \cdot \omega + c \cdot {\omega ^2}\;(a\ =\ 15.75,\;b\ =\ - 3.25\ {\rm{ }} \times\ {10^{ - 4}}{\,\,\rm{cm}}, c = 1.86 \times {10^{ - 9}}{\,\,\rm{c}}{{\rm{m}}^2})$ is used for fitting the experimental data around plasma resonance. If a linear function${\rm{\ }}\varepsilon _i^B( \omega )$ is utilized for Ni, the fitting results cannot be self-consistent.

Table 1 summarizes the parameters ${\tau _O},$${\omega _p}$, ${\omega _s}$, ${\varepsilon _c}( {{\omega _s}} )$, $\varepsilon _i^B( {{\omega _s}} )$ obtained by DLF-BE analysis and the zero-frequency relaxation time (${\tau _D}$) deduced from resistivity data for metals K, Rb, Ag, Au, Cs, Al and Ni. The comparative ${\omega _p}$ and $\varepsilon _i^B( \omega )$ results from DFT calculations using the Vienna Ab initio Simulation Package [31–33] and $\varepsilon _r^B( \omega )$ calculated from $\varepsilon _i^B( \omega )$ by Kramers-Kronig relations are also listed in Table 1.

**Table 1.** Drude's parameters of different metals obtained from various methods.

	Method	K [17]	Rb [18]	Cs [21]	Ag [20]	Au [20]	Al [25]	Ni [30]
$\frac{1}{{2\pi c{\tau _D}}}$ (cm^−1^)	DLF-BE	139 ± 5	202 ± 10	2230 ± 100	166 ± 50	236 ± 50	545 ± 50	523 ± 150
	DC	139	199	∼	144	188	520	398
$\frac{1}{{2\pi c{\tau _{AC}}}}$ (cm^−1^)	DLF-BE	1346	2734	4860	804	854	3836	98 566
$\frac{{{\omega _p}}}{{2\pi c}}$ (cm^−1^)	DLF-BE	34 408 ± 80	31 182 ± 70	26 960 ± 360	72 052 ± 1000	70 118 ± 1000	106 873 ± 1200	89 690 ± 5000
	DFT	34 048	30 817	24 752	74 627	75 188	106 383	64 103
$\frac{{{\omega _s}}}{{2\pi c}}$ (cm^−1^)	DLF-BE	30 899	27 778	24 108	31 006	20 325	121 007	75 820
	DFT	29 977	27 345	22 410	31 056	23 810	123 457	70 789
*λ*_p_ (nm)	DLF-BE	291	321	356	139	143	93	111
	DFT	294	324	404	134	133	94	156
	Drude [19]	283	313	352	139	138	79	—
*λ*_s_ (nm)	DLF-BE	324	360	415	323	492	82	132
	DFT	334	366	446	322	420	81	141
	Ref.	324 [17]	359 [18]	413 [21]	323 [23]	—	83 [25]	—
${\varepsilon _c}$ (${\omega _s}$)	DLF-BE	1.24 ± 0.005	1.26 ± 0.005	1.24 ± 0.03	5.4 ± 0.1	11.9 ± 0.1	0.78 ± 0.02	1.4 ± 0.1
	DFT	1.29	1.27	1.22	5.79	9.97	0.74	0.82
$\varepsilon _i^B$ (${\omega _s}$)	DLF-BE	0.054 ± 0.001	0.124 ± 0.001	0.25 ± 0.05	0.1 ± 0.02	0.1 ± 0.05	0.028 ± 0.002	1.82 ± 0.05
	DFT	0.06	0.13	0.32	0.3	0.57	0.03	2.1

DLF-BE: optical data processed with DLF analysis; DFT: modeled by DFT method; Drude: optical data fitted by Drude model; and DC: derived from $\frac{1}{{{\tau _D}}} = \frac{{\omega _p^2}}{{4\pi }}\ {\rho _{dc}}$ using resistivity data [28] and DFT calculated ${\omega _p}$.

As shown in Table 1, the screened plasma wavelength *λ*_s_ values obtained with the DLF-BE method agree perfectly with the experimental values. In the meantime, *λ*_p_ values from DLF-BE match well with the DFT calculations, in contrast to the previously reported discrepancies with the Drude model [19]. This confirms that the screening effect of bound electrons is well represented by a proper expression obtained from DLF-BE analysis. The zero frequency relaxation time (${\tau _D}$) from DLF-BE analysis is generally in good agreement with the results from DC electrical measurement [28] at room temperature for all the metals. However, for Cs, the $1/{\tau _D}$ obtained through DLF-BE analysis is significantly larger than the DC one, presumably because of differences in the sample impurity levels of Cs [21]. The DFT calculated${\rm{\ }}{\varepsilon _c}( {{\omega _s}} )$ and $\varepsilon _i^B$ are also consistent with the parameters derived from experimental data. We noted that for alkali metals, while the elastic polarization effects are relatively small (${\omega _p}/{\omega _s}$ ∼1.1–1.2), the inelastic polarization effects are very large, that is $1/{\tau _{AC}}$ values are 10–15 times higher than $1/{\tau _D}$. For transition metals Ag, Au and Ni, the elastic polarization effects are much larger (2–4 times), while the inelastic polarization effects are moderately larger (∼4 times). In short, the $1/{\tau _{AC}}$ term contributes significantly more than $1/{\tau _D}$ in ${\tau _o}( {{\omega _s}} )$ in all cases here. This suggests that the bound electron effect has a dominant contribution for electron quantum relaxation in the UV-visible optical frequency range, and also induces large changes in plasma resonance frequencies. On the other hand, the results also indicate that the historical assumption of frequency-independent quantum relaxation time in the Drude term cannot describe the optical response correctly. This is the first time that bound electron polarization effect has been used to determine quasi-particle effective properties—carrier density and quantum relaxation time—of conduction electrons.

**Figure 6.**
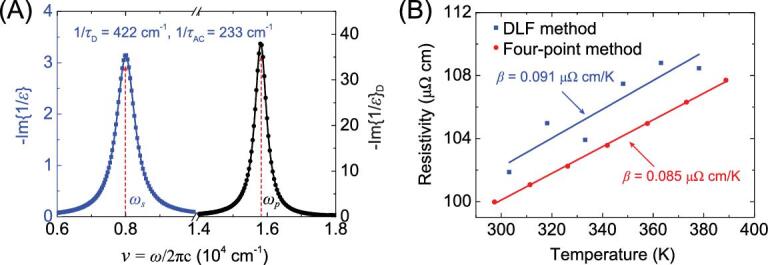
(A) DLF-BE analysis of ITO at 303 K. (B) Comparison of resistivity ${\rho _D}( T )$ from the optical method and four-point probe method.

We further explore application of DLF-BE analysis to non-metals. To test the validity of this method to conducting semiconductors, a 176 nm thick indium-tin oxide (ITO) film sample was measured by ellipsometry at 303 K. The resistivity ${\rho _D}$ obtained by DLF-BE in Fig. 6(A) is 101.9 $\mu {\rm{\Omega }} \cdot {\rm cm}$, matching well with the DC four-point probe measurement of 100.4 $\mu {\rm{\Omega }} \cdot {\rm cm}$ at similar temperature. This result proves that the method can be applied successfully in semiconducting materials with conduction electrons.

According to Matthiessen's rule, $1/{\tau _D}$ is composed of two terms:

(14) \begin{equation*}1/{\rm{\ }}{\tau _D} = {\rm{\ }}1/{\tau _{e - i}} + 1/{\tau _{e - p}}\left( T \right).\end{equation*}

Here $1/{\tau _{e - i}}$ represents the scattering rate of electron-impurity (extrinsic) and $1/{\tau _{e - p}}( T )$, the scattering rate of electron-phonon (intrinsic), which is temperature-dependent.

To further separate the two terms, temperature-dependent measurements of dielectric constants were required. Ellipsometry measurement was carried out on ITO film from 303 K to 378 K at 15 K intervals. The results of DLF-BE analysis are given in Table 2. The sheet resistance was also measured from 297.3 K to 388.8 K at ∼15 K intervals by four-point probe. Figure 6(B) compares the resistivity of the ITO sample obtained with DLF-BE analysis of optical data with the four-point probe data at various temperatures. Using the relation ${\rho _{dc}} = \frac{{4\pi }}{{\omega _p^2}}\ \ \frac{1}{{{\tau _D}}} = {\rho _{e - i}}\ + \beta \cdot T$, the value of resistivity originated from electron-impurity scattering was determined by a non-geometry-sensitive optical method to be ${\rho _{e - i}} = \ 74.8{\rm{\ }}$$\mu {\rm{\Omega }} \cdot {\rm cm}$, nearly the same as the geometry-sensitive DC contact measurement value of $74.7{\rm{\ }}$$\mu {\rm{\Omega }} \cdot {\rm cm}$. Meanwhile, the deviation of the temperature-dependence slope *β* of the electron-phonon term obtained by the two methods agrees well (<10%) considering the four-point probe method is dependent on a geometry factor with limited accuracy. The results seem to suggest that our method is applicable to electrical transport measurement of both metals and semiconductors with conduction electrons through impurity and optical doping at various temperatures, providing a potentially fast, non-destructive and micro-area detection method for semiconductor industrial applications.

**Table 2.** Fitting parameters of ITO obtained at different temperatures.

Parameters	303 K	318 K	333 K	348 K	363 K	378 K
$\frac{1}{{2\pi c{\tau _D}}}$ (cm^−1^)	422	434	430	444	449	447
$\frac{1}{{2\pi c{\tau _{AC}}}}$ (cm^−1^)	233	235	237	239	241	243
$\frac{{{\omega _p}}}{{2\pi c}}$ (cm^−1^)	15 782.4	15 766.6	15 773.6	15 760.7	15 752.6	15 741.7
$\frac{{{\omega _s}}}{{2\pi c}}$ (cm^−1^)	7980.6	7971.9	7975.7	7968.4	7964.0	7958.6
${\varepsilon _c}$ (${\omega _s}$)	3.9	3.9	3.9	3.9	3.9	3.9
$\varepsilon _i^B$ (${\omega _s}$)	0.114	0.115	0.116	0.117	0.118	0.119
${\rho _D} \cdot (\mu {\rm{\Omega }} \cdot {\rm cm})$	101.9	105.0	103.9	107.5	108.8	108.5

Thus, we have demonstrated that the large discrepancies in the electrical transport properties between the Drude–Sommerfeld model and DC contact measurements in metallic elements can be resolved by DLF-BE analysis. The bound electron contributions result in an extra damping effect of conduction electrons at plasma resonance and a shift of plasma resonance frequency. From a physics point of view, the optical radiation should also interact with the background lattice, where the atoms are surrounded (or screened) by bound electrons to cause polarization (bound electron cloud deformation), which in turn affects the conduction electrons. The elastic deformation screens conduction electron charge, leading to a change in effective carrier density and a shift of the plasma resonance. The inelastic deformation causes additional scattering/loss in conduction electron movement and reduces quantum relaxation time.

## CONCLUSION

In this study, by adopting DLF analysis into the physics of plasma resonance, the reciprocal quantum relaxation time in DC field $1/{\tau _D}$ and at non-zero frequency $1/{\tau _{AC}}$ is directly measured for the first time through a damping effect of plasma resonance. The DLF-BE analysis results are consistent with various experimental results and theoretical calculations. The results show that bound electron inelastic scattering to conduction electrons is the dominating damping effect of quantum relaxation time at optical frequencies. Although bound electron contributions to dielectric functions have been known for a long time, the contribution to quantum relaxation time of conduction electrons has never been realized until now.

## METHODS

### Calculations of ${{\boldsymbol\varepsilon ^{\boldsymbol B}}}(\boldsymbol \omega )$ and ${\boldsymbol \omega _{\boldsymbol p}}$ using DFT

The DFT calculations were carried out with the Perdew–Burke-Ernzerhof exchange-correlation function using the Vienna Ab initio Simulation Package (VASP) [31–33]. The plane-wave energy cutoff was set to 300–428 eV depending on the systems, and projector augmented-wave pseudopotentials were used. For the transition metals Ni, Ag and Au, the Hubbard U method was utilized with effective U−J values of 3.5, 2.8 and 3.2 eV, respectively. Monkhorst-Pack *k*-point grids were used for sampling the Brillouin zone, with spacing of ∼0.03 Å^−1^. The imaginary dielectric function of bound electrons was calculated following Fermi's golden rule under the dipole approximation [34], as shown in Equation (15).

(15) \begin{eqnarray*}\varepsilon _i^B(\omega ) &=& \left(\frac{1}{{4\pi {\varepsilon _0}}}\right){\left(\frac{{2\pi e}}{{m\omega }}\right)^2}{\sum\nolimits_{k,c,v}}\nonumber\\ &&\times {{\left| {\left\langle {{\rm{\Psi }}_k^c\left| {{{\bf e}} \cdot {{\bf p}}} \right|{\rm{\Psi }}_k^v} \right\rangle } \right|} ^2}\delta \left(E_k^c - E_k^v - \hbar \omega \right) ,\nonumber\\ \end{eqnarray*}

where **e** is the polarization vector of the incident electric field, **p** is the momentum operator, and *c* and *v* represent the conduction and valence bands, respectively. The real dielectric function of bound electrons $\varepsilon _r^B( \omega )$ was then obtained from $\varepsilon _i^B( \omega )$ through the Kramers-Kronig relation. ${\omega _p}$ was obtained through direct-current electrical conductivity calculation using the Boltzmann transport equation (Equation (16)), as implemented in the BoltzTraP2 program [35]

(16) \begin{eqnarray*}\omega _p^2 &=& \ 4\pi {\sigma _{dc}}/\ {\tau _{dc}} = \frac{{{e^2}}}{{2{\pi ^2}}}\ \int\!\!\!\int \sum\nolimits_n {\frac{{\partial {E_{n,{\boldsymbol{k}}}}}}{{\partial {\boldsymbol{k}}}}}\nonumber\\ && \otimes {\frac{{\partial {E_{n,{\boldsymbol{k}}}}}}{{\partial {\boldsymbol{k}}}}} \ \left( { - \frac{{\partial f\left( {E,T} \right)}}{{\partial E}}} \right)\delta \left( {E - {E_{n,{\boldsymbol{k}}}}} \right)\nonumber\\ &&\times\ d{\boldsymbol{k}}dE, \end{eqnarray*}

where ${E_{n,\! {\boldsymbol{k}}}}$ is the orbital energy calculated using VASP and *f* the Fermi-Dirac distribution. Then ${\omega _{s - DFT}}$ can be estimated by ${\omega _s} = {\omega _p}/\sqrt {1 + \varepsilon _r^B( {{\omega _{s - exp}}} )} $.

### ITO measurements

ITO films of nominal thickness of 180 nm were purchased from Hefei Kejing Material Technology Co., Ltd., prepared by magnetron sputtering. Spectra of the ellipsometric angles ψ (amplitude ratio) and Δ (phase shift difference) were acquired at various temperatures with a commercial spectroscopic ellipsometer (RC2, J. A. Woollam) operating in reflection mode in the 210–2500 nm wavelength range. Focusing probes were used to reduce the beam diameter to 500 μm at the sample surface. All the measurements were performed at incidence angle of 70°. The complex dielectric function calculated from ψ and Δ was achieved using CompleteEASE software, with surface roughness considered. The refractive index n and the extinction coefficient k of ITO parameterized @632.8 nm at room temperature are 1.740 and 0.033, respectively. A standard heat stage (HTC-100) was used to control the temperature. Rate of temperature change was slow enough (0.5 K/minute) to ensure the cooling and heating data were consistent for more accurate temperature measurement.

## Supplementary Material

nwaa242_Supplemental_File
