# Supplementary material for: A direct measurement method of quantum relaxation time
Source: Natl Sci Rev. 2020 Sep 18;8(4):nwaa242. doi: 10.1093/nsr/nwaa242 (PMC8288436; doi:10.1093/nsr/nwaa242)
Supplement: nwaa242_Supplemental_File [file nwaa242_supplemental_file.docx]

Supplementary Information

A Direct Measurement Method of Quantum Relaxation Time

**S1. The data processing procedure**


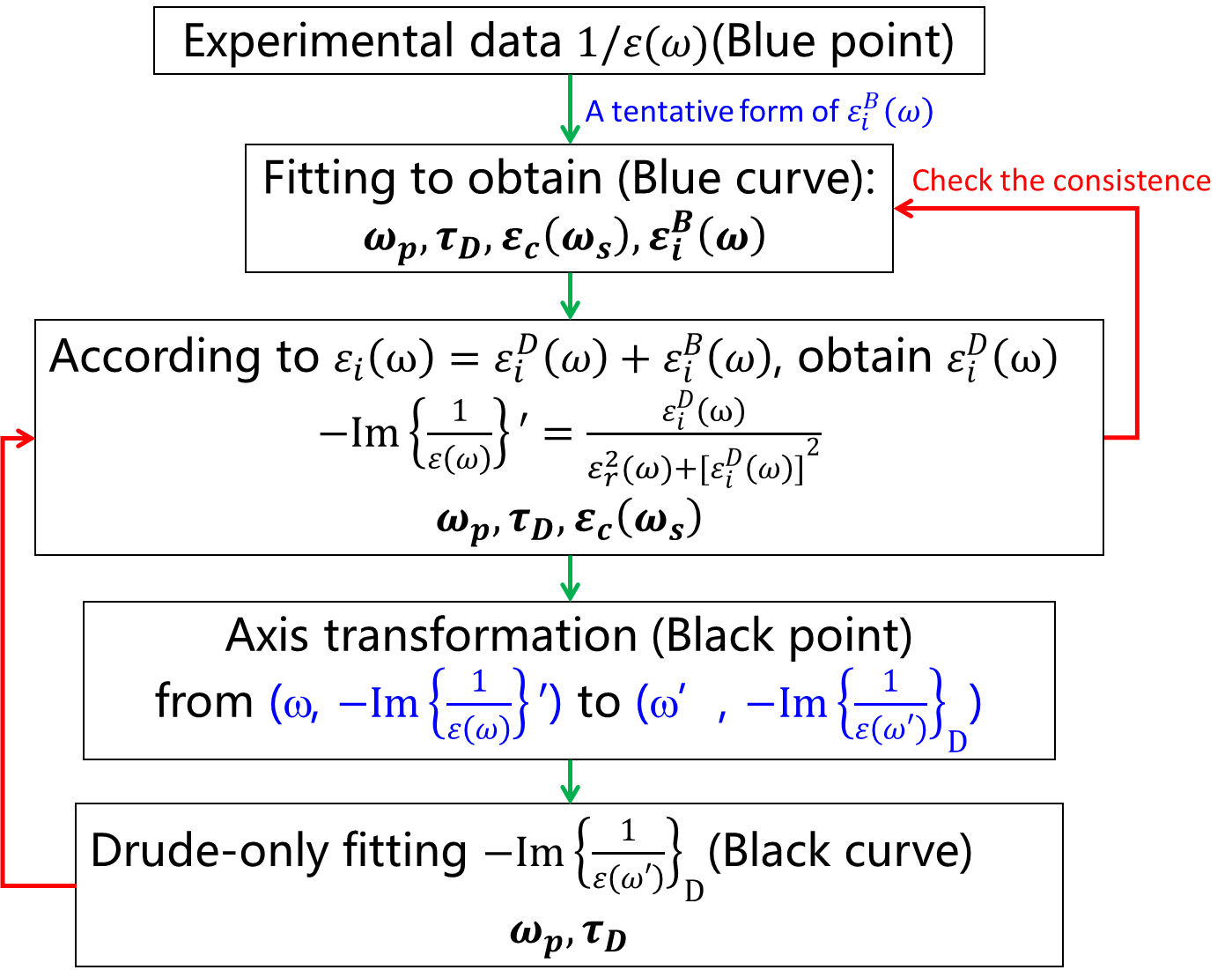


**Supplementary Figure S1** The data processing procedure.

First, a tentative form of $\varepsilon_{i}^{B}\left( \omega\right)$ around the plasma frequency is given based on the asymmetry of the resonance peak (blue points in **Fig. 2**). Using the equation:

$-Im\left( \frac{1}{\varepsilon\left( \omega\right)} \right)=\frac{\frac{\omega_{p}^{2}}{\omega\tau_{D}\left( \omega^{2}+\tau_{D}^{-2} \right)}+\varepsilon_{i}^{B}\left( \omega\right)}{\left( \varepsilon_{c}\left( \omega_{s} \right)-\frac{\omega_{p}^{2}}{\omega^{2}+\tau_{D}^{-2}} \right)^{2}+\left( \frac{\omega_{p}^{2}}{\omega\tau_{D}\left( \omega^{2}+\tau_{D}^{-2} \right)}+\varepsilon_{i}^{B}\left( \omega\right) \right)^{2}}$ (1)

to fit the peak (blue curves in **Figs. 2&3**) could yield $\omega_{p}$, $\tau_{D}$, $\varepsilon_{c}\left( \omega_{s} \right)$, and $\varepsilon_{i}^{B}\left( \omega\right)$. Since $\varepsilon_{r}^{B}\left( \omega\right)$ is nearly a constant around the plasma frequency, we could assume $\varepsilon_{c}\left( \omega_{s} \right)=1+\varepsilon_{r}^{B}\left( \omega\right)$. Then according to $\varepsilon\left( \omega\right)=\varepsilon^{D}\left( \omega\right)+\varepsilon^{B}\left( \omega\right)$, we could obtain the Drude term $\varepsilon_{i}^{D}\left( \omega\right)$, and **Eq. (1)** changes into

$-\mathrm{Im}\left\{ \frac{1}{\varepsilon\left( \omega\right)} \right\}^{'}=\frac{\varepsilon_{i}^{D}\left( \omega\right)}{\varepsilon_{r}^{2}\left( \omega\right)+\left[ \varepsilon_{i}^{D}\left( \omega\right) \right]^{2}}=\frac{\omega_{s}^{2}\omega/\tau_{D}}{\varepsilon_{c}\left( \omega_{s} \right)\left[ {(\omega^{2}-\omega_{s}^{2})}^{2}+\omega^{2}\tau_{D}^{-2} \right]}$, (2)

yielding $\omega_{p}$, $\tau_{D}$, $\varepsilon_{c}\left( \omega_{s} \right)$ again. In order to show a pure and intuitive Drude contribution of dielectric loss, we further make an axis transformation from $(\omega,-\mathrm{Im}\left\{ \frac{1}{\varepsilon\left( \omega\right)} \right\}^{'})$ to ${(\omega^{'}, -Im\left\{ \frac{1}{\varepsilon\left( \omega' \right)} \right\}}_{D})$ (black points in **Fig. 2**), where the axis shift from $\omega$ to $\omega^{'}$ is based on the difference between $\omega_{p}$ and $\omega_{S}$. By using the equation:

${-Im\left\{ \frac{1}{\varepsilon\left( \omega\right)} \right\}}_{D}=\frac{\omega_{p}^{2}\omega/\tau_{D}}{\left( \omega^{2}-\omega_{p}^{2} \right)^{2}+\omega^{2}\tau_{D}^{-2}}$, (3)

with a similar mathematical form of $-\mathrm{Im}\left\{ \frac{1}{\varepsilon\left( \omega\right)} \right\}^{'}$, to fit the Drude term, we could also determine $\tau_{D}$ (black curves in **Fig. 2**). If the $\tau_{D}$ obtained from the three fittings are very close to each other, we think these fitting results are reliable.

**S2. The real and imaginary parts of dielectric function for bound electrons** $\boldsymbol{\varepsilon}_{\boldsymbol{r}}^{\boldsymbol{B}}\boldsymbol{(\omega)}$ **and** $\boldsymbol{\varepsilon}_{\boldsymbol{i}}^{\boldsymbol{B}}\boldsymbol{(\omega)}$ **of 7 metals**


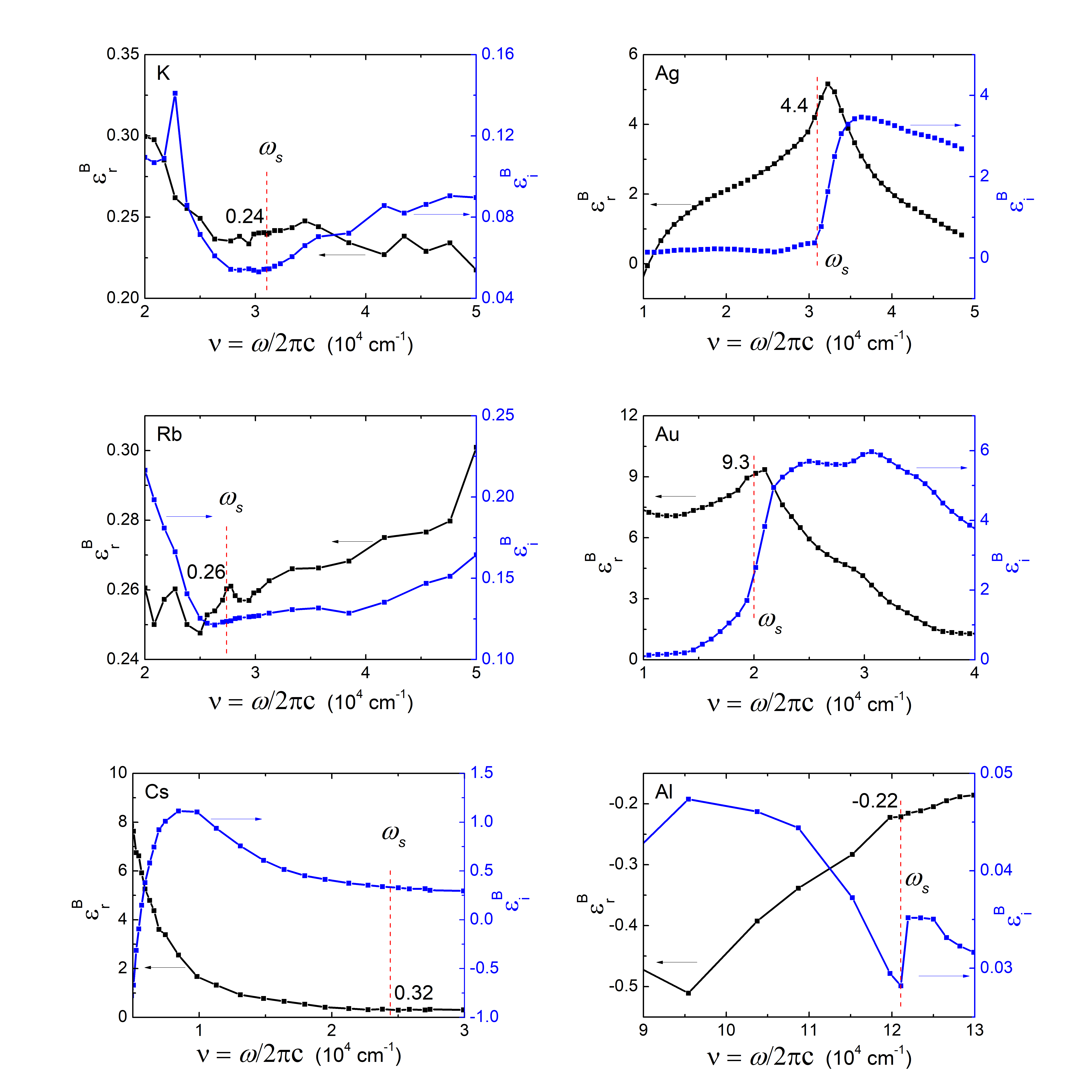


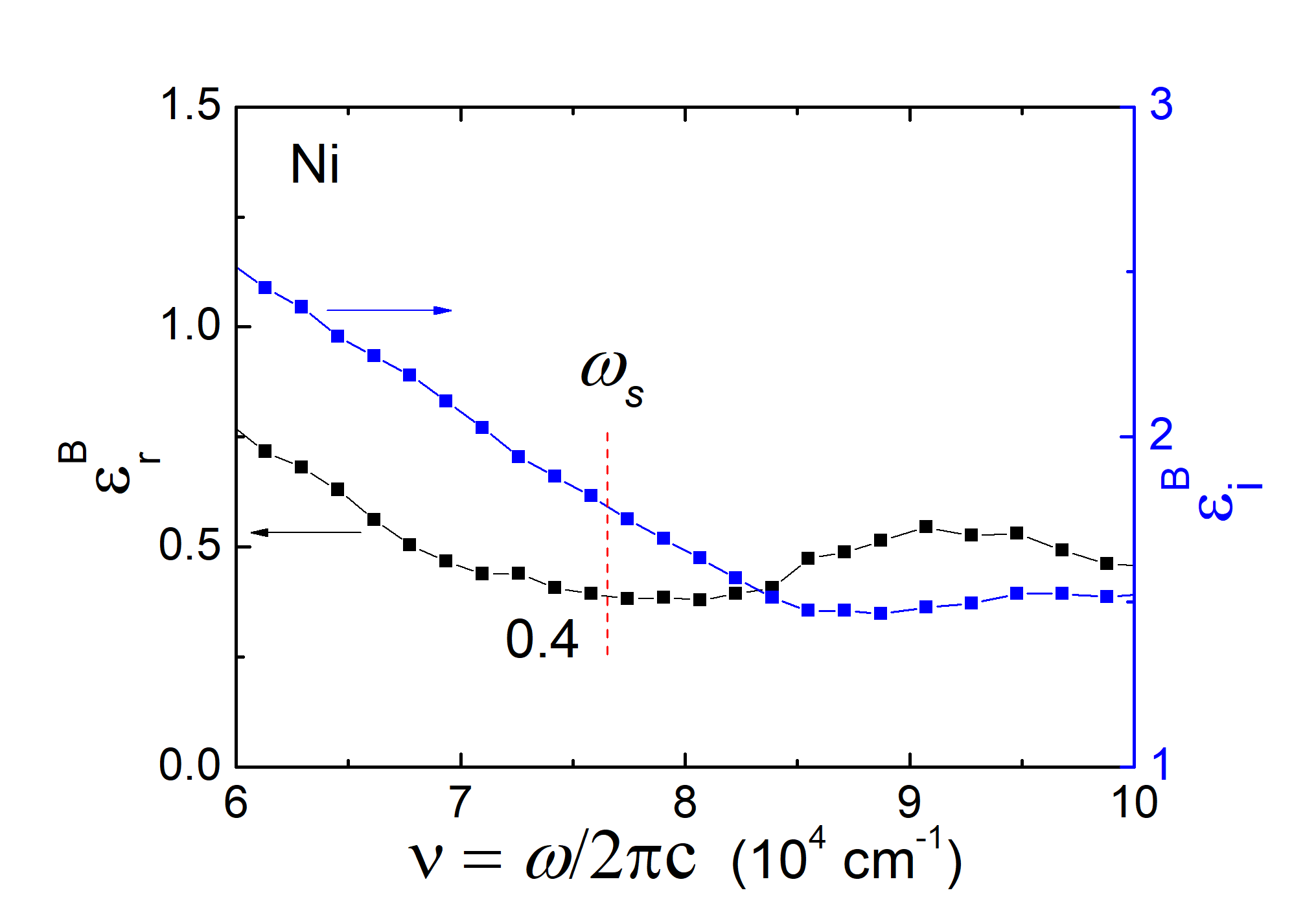


**Supplementary Figure S2** The real and imaginary parts of dielectric function for bound electrons $\varepsilon_{r}^{B}(\omega)$ and $\varepsilon_{i}^{B}(\omega)$ of 7 metals were extracted by the relation: $\varepsilon^{B}(\omega)=\varepsilon^{exp}(\omega)-\varepsilon^{D}$. The optical data are used the same as **Figs. 2&3** and the Drude parameters are shown in **Table 1**.
